# Pancreatitis

**DOI:** 10.1111/papr.70089

**Published:** 2025-10-15

**Authors:** Alaa Abd‐Elsayed, Christopher Gilligan

**Affiliations:** ^1^ Department of Anesthesiology University of Wisconsin School of Medicine and Public Health Madison Wisconsin USA; ^2^ Robert Wood Johnson University Hospital New Brunswick New Jersey USA

Chronic pancreatitis is a condition in which there are recurrent inflammatory episodes that result in the replacement of the pancreatic parenchyma by fibrous tissue. Pancreatitis can lead to severe pain that can be challenging to treat.

A literature review conducted by Zeggeren et al. [1] aimed at describing the diagnosis and treatment options for chronic pancreatitis.

The review found a broad range of how chronic pancreatitis is being managed. Options start with medication management, surgery, and interventional pain procedures. Procedures may include radiofrequency ablation of the splanchnic nerves, spinal cord stimulation, and celiac plexus blocks.

The authors concluded that the management of chronic pancreatitis is complex and requires a multidimensional and individualized approach. There is a need for randomized controlled trials to test the efficacy of different interventions.

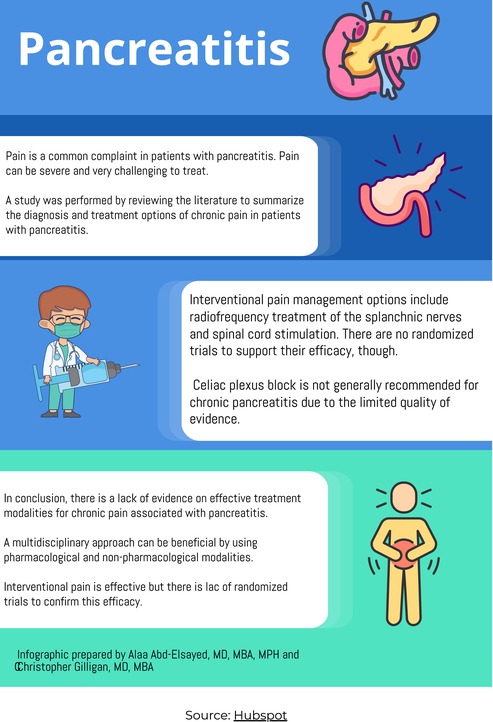

## Conflicts of Interest

Dr. Christopher Gilligan is the editor in chief of *Pain Practice* and Dr. Alaa Abd‐Elsayed is a section editor of *Pain Practice*.

## Data Availability

Data sharing not applicable to this article as no datasets were generated or analyzed during the current study.
